# Residual Stress in Epoxy-Based Insulators: Formation, Detection, and Reliability

**DOI:** 10.3390/molecules31142410

**Published:** 2026-07-08

**Authors:** Jin Li, Siyuan Chen, Hucheng Liang, Boxue Du

**Affiliations:** State Key Laboratory of Smart Power Distribution Equipment and System, Tianjin University, Tianjin 300072, China; lijin@tju.edu.cn (J.L.); chensiyuan@tju.edu.cn (S.C.); duboxue@tju.edu.cn (B.D.)

**Keywords:** GIL, epoxy-based insulator, residual stress, detection method, mechanical reliability

## Abstract

Gas-insulated switchgears (GISs) and gas-insulated transmission lines (GILs) are essential for large-capacity power transmission in demanding environments, such as high drops, large spans, and heavy pollution. As the core components providing both electrical insulation and mechanical support, ultra-high voltage (UHV) epoxy-based insulators often suffer from high internal residual stress. This issue, compounded by a lack of reliable detection methods, frequently results in equipment being commissioned with hidden defects. To address this, this review first examines the formation mechanisms of curing deformation and residual stress in oversized insulators based on cure kinetics and thermo-chemical coupling models. Subsequently, it provides a comprehensive summary of current residual stress measurement techniques, comparing the applicability and limitations of embedded sensors, direct mechanical measurements, and indirect non-destructive testing (NDT) methods. Finally, by coupling residual stress with filler sedimentation, the stress distribution patterns and mechanical reliability of epoxy-based insulators across different life-cycle stages are analyzed. These insights offer valuable theoretical references for the structural design, process optimization, and performance evaluation of oversized epoxy-based insulators, ultimately contributing to the intrinsic safety of UHV power equipment.

## 1. Introduction

Ultra-high voltage (UHV) transmission corridors from energy bases face extreme environmental challenges, such as high altitudes, large drops, and variable climates. Meanwhile, the construction of alternating-current (AC) main grids in load centers must cross special areas like rivers and lakes. Therefore, it is urgent to overcome the limitations of traditional transmission corridors [1,2]. Gas-insulated switchgears (GISs) and gas-insulated transmission lines (GILs) are metal-enclosed high-voltage apparatuses in which current-carrying conductors and insulating gas are sealed inside grounded enclosures. GIS is mainly used for compact substations and switching equipment, whereas a GIL is used for long-distance and large-capacity power transmission. They possess strong environmental adaptability and small footprints, making them attractive solutions for large-capacity (GW level, carrying current > 8000 A) transmission under these special conditions [3,4,5]. Advanced polymeric dielectrics and composite insulating materials have been increasingly developed for high-performance electrical equipment [6,7].

Epoxy-based insulators, such as GIS basin-type insulators and GIL tri-post insulators, are core components of the transmission systems. They provide both electrical insulation and mechanical support. Their safety directly affects the stable operation of the UHV transmission system [8,9,10]. Nearly 60,000 insulators are required per 100 km of double-circuit AC GIL, and the failure of any single insulator may interrupt the operation of the entire transmission line. Taking China as an example, Table 1 shows several typical fault types of operating epoxy-based insulators in recent years [11]. During factory tests, faults such as insulator fracture may occur. For example, during the GIL crimping process, wedging the wedge ring can cause cracking at the bonding edge between the insulator and the sleeve. During the handover and operation stages, insulator faults are mainly burst failures. Following a failure, a single leg of the insulator is typically severely damaged and scattered as fragments throughout the pipeline, while the metal inserts remain secured to the enclosure shell. Furthermore, carbonized ablation channels are observed on the surface or within the insulator body near the conductor rod [12,13].

Analysis shows that these sudden faults are closely related to the internal stress level of the insulators. Especially during the manufacturing process, large residual stress may be generated in the insulator due to high-temperature curing and processing. High residual stress not only affects the mechanical properties of the material but may also become a hidden danger for faults. An oversized epoxy-based insulator has a diameter close to one meter, which leads to a continuously high internal forming stress. This makes the insulator highly vulnerable to crack propagation and failure during commissioning, assembly, and operation, ultimately leading to severe burst faults. Due to the lack of effective stress detection methods, these residual stresses cannot be monitored and evaluated in time. As a result, support insulators may carry hidden dangers during operation, directly threatening equipment safety. Therefore, accurately understanding the formation mechanism of residual stress in epoxy-based insulators, developing new detection technologies, and achieving accurate residual stress evaluation are key challenges that urgently need to be solved for large-scale UHV GIS/GIL equipment.

This work is intended as a narrative review. Relevant studies up to 2026 were collected from Web of Science, Scopus, IEEE Xplore, ScienceDirect, SpringerLink, and CNKI using keywords related to epoxy insulators, GIS/GIL, residual stress, curing shrinkage, thermo-chemical modeling, alumina sedimentation, stress detection, and mechanical reliability. Studies with limited relevance to electrical insulation or residual stress were excluded.

Existing studies generally address curing-induced stress, residual-stress measurement, or mechanical reliability separately, while their interactions with filler sedimentation throughout the insulator life cycle have received limited systematic attention. Compared with previous studies, this review focuses on oversized epoxy/Al_2_O_3_ insulators for UHV GIS/GIL equipment and integrates three closely related aspects: (i) cure kinetics and thermo-chemical-mechanical formation mechanisms of residual stress; (ii) residual-stress detection technologies, including embedded sensors, direct mechanical measurements, and indirect non-destructive testing (NDT) methods; and (iii) mechanical reliability evaluation considering residual stress and filler sedimentation during manufacturing, assembly, and operation. This study provides theoretical support for improving the manufacturing quality and operational safety of epoxy-based insulators.

## 2. Formation Mechanism of Residual Stress

The curing process of epoxy resin-based composite insulating materials is complex. Limited by heat transfer capabilities, the internal curing reaction is asynchronous, which easily leads to internal stress gradients within the components. Furthermore, the residual stress in the components is exacerbated by several factors: the chemical shrinkage caused by the internal curing reaction, the thermal shrinkage during the cooling stage, and the mismatch of material parameters between the composite and the mold. Therefore, to improve product quality, researchers have conducted extensive studies in recent years on predicting the residual stress and deformation degree of cured epoxy resin products reasonably and effectively [14,15].

### 2.1. Cure Kinetics Model

**Figure 1:**
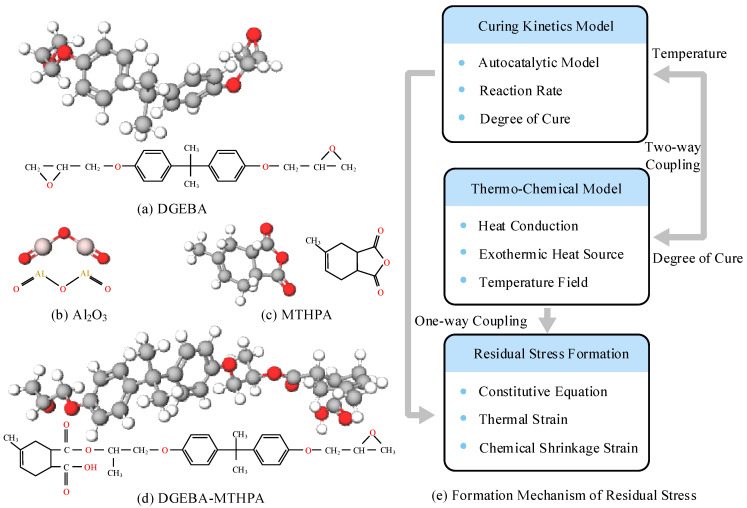
Molecular structures of the epoxy composite and the formation mechanism of residual stress: (**a**) DGEBA, (**b**) Al_2_O_3_, (**c**) MTHPA, (**d**) DGEBA-MTHPA, (**e**) Formation mechanism of residual stress.

Analyzing the residual stress of epoxy composites is fundamentally based on determining their curing reaction characteristics. Based on the reaction type, an appropriate kinetic model is selected for the specific formulation of the insulators. During the preparation of epoxy-based insulators, the relevant reaction of the epoxy composite insulating material refers to the reaction between the epoxy resin and the curing agent to form a three-dimensional cross-linked network structure [16,17]. As shown in Figure 1, a typical composite system consists of bisphenol-A epoxy resin, alumina fillers, and an anhydride curing agent. The cross-linking of these molecular structures drives the cure kinetics, which involves complex physical processes and chemical reactions. Studying the cure kinetics helps clarify the reaction behavior of the epoxy/alumina system at different curing stages, such as the reaction rate and characteristic mechanisms at various curing stages. Based on different treatment methods for apparent activation energy, existing research methods can be divided into model-fitting and non-model-fitting methods. The model-fitting method can be further classified into phenomenological models and mechanistic models. Phenomenological models express the basic equations of cure kinetics using parameters. They do not involve detailed reaction products or mechanisms, offering better engineering applicability. Consequently, among phenomenological models, the n-order reaction model and the autocatalytic model are currently the most widely and maturely applied in studying the cure kinetics of epoxy composites [18,19].

Thermal analysis methods can accurately characterize the changes in thermodynamic properties of the tested samples during the curing process. Among them, differential scanning calorimetry (DSC) is widely used in the curing reaction kinetics research of epoxy composites [20,21]. Omrani et al. conducted non-isothermal DSC experiments on the DGEBA/nano-alumina composite system, fitted the curing kinetic model using the Avrami equation, and verified the catalytic effect of nano-fillers on the epoxy curing process [22]. Bi et al. [23] studied the effects of different curing agent contents on the curing reaction process of the epoxy composite insulation system. The results show that the actual curing process of the epoxy resin/Al_2_O_3_ system conforms to the Sestak–Berggren (m, n) model, and the autocatalytic reaction plays a dominant role during curing [23]. Wang et al. calculated the reaction activation energy of the epoxy/Al_2_O_3_ composite system according to the Kissinger method. The model fitted using the Malek method corresponds well with the experimental results [24].

### 2.2. Thermo-Chemical Model

The exothermic curing reaction produces non-uniform temperature and degree-of-cure fields in large epoxy-based insulators. Residual stress prediction therefore requires coupling the thermo-chemical model with an appropriate mechanical constitutive model [25,26]. Kim et al. established a viscoelastic equation for AS4/3501 prepregs and compared the effects of factors such as chemical shrinkage and thickness on residual stress during the curing of laminates [27]. Yuan et al. proposed a multi-scale prediction model. They established a coupled model containing thermochemistry and residual stress at the macro scale and introduced the viscoelastic constitutive equation of the resin at the micro scale [28]. Puentes et al. [29] proposed a method based on the sandwich beam theory to continuously capture modulus development during the curing of thermosetting resins. They further compared the applicability of various constitutive models, such as those by Bogetti and Johnston, in characterizing the relationship between the degree of cure and elastic modulus [29]. The accuracy of these constitutive models depends strongly on the availability of cure- and temperature-dependent material properties. In practice, complete data are difficult to obtain, and simplified or extrapolated parameters may introduce uncertainty into the predicted stress [30].

Most existing residual stress studies on epoxy composites concern fiber-reinforced systems, whereas GIS/GIL insulators are commonly manufactured from particulate-filled epoxy/Al_2_O_3_ composites. Chen et al. [31] established a coupled temperature–cure–mechanical model for an 1100 kV GIS basin-type insulator. The highest stress occurred near the central insert, and chemical shrinkage during curing and thermal shrinkage during cooling were identified as the main stress sources [31].

During the curing of epoxy-based insulators, the process environment temperature affects the curing reaction of the epoxy composite insulating material by increasing the internal temperature of the insulator. Meanwhile, the degree of cure can characterize the chemical conversion rate of the epoxy resin. At this stage, the cross-linking reaction between the epoxy resin and the anhydride releases heat, which further affects the internal temperature of the insulator. Therefore, the thermo-chemical model of the epoxy composite insulating material is essentially a three-dimensional heat conduction model and a curing kinetic model containing a nonlinear internal heat source caused by the exothermic chemical reaction of the resin matrix. According to Fourier’s law of heat conduction [32,33]

(1) $$\rho_{c}C_{p}\frac{\partial T}{\partial t}=\frac{\partial }{\partial x}(k_{xx}\frac{\partial T}{\partial x})+\frac{\partial }{\partial y}(k_{yy}\frac{\partial T}{\partial y})+\frac{\partial }{\partial z}(k_{zz}\frac{\partial T}{\partial z})+Q_{c}$$

where *T* is the absolute temperature (K), *t* is time (s), $\rho_{c}$ is the effective density of the epoxy composite (kg/m^3^), *C*_p_ is the effective specific heat capacity (J/(kg·K)), *Q*_c_ is the volumetric heat-generation rate caused by the curing reaction (W/m^3^), and *k*_xx_, *k*_yy_, and *k*_zz_ are the thermal conductivities of the material in different directions (W/(m·K)). The existing degree of alumina sedimentation has a minor effect on the thermal conductivity of the composite. Therefore, the thermal conductivity of the epoxy/Al_2_O_3_ composite insulating material can be considered isotropic, meaning *k*_xx_ = *k*_yy_ = *k*_zz_. The magnitude of the internal heat source is related to the curing rate and can be expressed by (2) [34]

(2) $$Q_{c}=\rho_{c}\Delta H_{R}\frac{\partial \alpha }{\partial t}$$

where Δ*H*_R_ is the total heat of reaction released during the curing of the epoxy composite (J/kg), and *α* is the degree of cure of the material, which can be obtained by (3) [35]

(3) $$\alpha (t)=\frac{\Delta H_{t}}{\Delta H_{R}}$$

where Δ*H*_t_ is the heat released by the reaction of the epoxy composite insulating material up to time *t* (J/kg), and Δ*H*_R_ is the total reaction enthalpy obtained after complete curing on the same mass basis. Therefore, α is dimensionless and varies from 0 to 1.

The term ∂*α*/∂*t* is the curing rate of the material at time t, expressed by (4)

(4) $$\frac{\partial \alpha }{\partial t}=f(\alpha ,T)$$

where *f* (*α*, *T*) is the curing-rate function with the unit s^−1^, whose specific form is determined by the selected kinetic model.

### 2.3. Curing Deformation and Residual Stress Formation

By coupling the thermo-chemical model with the cure kinetics model of the epoxy composite insulating material, the specific distribution of temperature and degree of cure at any position during the preparation of the epoxy-based insulator can be determined at any given time. The internal residual stress of the insulator mainly includes the thermal stress caused by the temperature variation in the epoxy composite and the stress induced by chemical shrinkage during the resin cross-linking reaction. Therefore, the established residual stress model must be based on the spatiotemporal variations in temperature and degree of cure during the curing process.

For the composite insulating material used in epoxy-based insulators, a linear elastic stress–strain model can be applied to determine the residual stress distribution after curing. The constitutive equation considering both thermal strain and chemical shrinkage strain is as follows [36]:

(5) $$\left\{\sigma \right\}=[C]\left\{\varepsilon_{c}-\varepsilon_{0}^{T}-\varepsilon_{0}^{r}\right\}+\left\{\sigma_{0}\right\}$$

where {*σ*} and {*σ*_0_} are the internal stress and initial stress (Pa), respectively; {*ε*}, {$\varepsilon_{0}^{T}$}, and {$\varepsilon_{0}^{r}$} are the total, thermal, and chemical-shrinkage strain vectors, respectively; and [C] is the constitutive stiffness matrix (Pa). All strain components are dimensionless. This formulation is computationally efficient, but it cannot reproduce the time-dependent stress relaxation of the resin during curing and may therefore overestimate the retained stress. Thermo-viscoelastic models provide a more complete description, although they require additional relaxation data over a wide range of temperatures and degrees of cure.

For the internal strain of the epoxy composite insulating material, the thermal strain and chemical shrinkage strain can be simply summed. The thermal strain can be obtained by (6) [31]

(6) $$\varepsilon_{0}^{T}=\alpha_{T}(T-T_{\mathrm{ref}})$$

where *α_T_* is the thermal expansion coefficient of the material (1/K), and *T_ref_* is the reference temperature (K).

The chemical shrinkage strain can be derived from the relative volumetric shrinkage of the material. For a representative material element with initial dimensions *l*_1_, *l*_2_, and *l*_3_, the curing-induced volume change is [37]

(7) $$\Delta V=\Delta l_{1}l_{2}l_{3}+l_{1}\Delta l_{2}l_{3}+l_{1}l_{2}\Delta l_{3}-\Delta l_{1}\Delta l_{2}l_{3}-l_{1}\Delta l_{2}\Delta l_{3}-\Delta l_{1}l_{2}\Delta l_{3}+\Delta l_{1}\Delta l_{2}\Delta l_{3}$$

where Δ*l*_1_, Δ*l*_2_, and Δ*l*_3_ are the reductions in the three dimensions during curing, and *V* = *l*_1_*l*_2_*l*_3_ is the initial volume of the material element.

Meanwhile, the relative volumetric shrinkage of the micro-element can be obtained from the strains in the three directions:

(8) $$\Delta v_{r}=\frac{\Delta V}{V}=\varepsilon_{1}+\varepsilon_{2}+\varepsilon_{3}-\varepsilon_{1}\varepsilon_{2}-\varepsilon_{2}\varepsilon_{3}-\varepsilon_{1}\varepsilon_{3}+\varepsilon_{1}\varepsilon_{2}\varepsilon_{3}$$

where $\Delta v_{r}$ is the relative volumetric shrinkage, and $\varepsilon_{1}$, $\varepsilon_{2}$ and $\varepsilon_{3}$ are the dimensionless linear shrinkage strains in the three principal directions. Assuming approximately equal macroscopic shrinkage in the three principal directions, *ε*_1_ ≈ *ε*_2_ ≈ *ε*_3_, the linear shrinkage strain is [38]

(9) $$\varepsilon_{0}^{r}=1-\sqrt[{3}]{1-\Delta v_{r}}$$

The relative volumetric shrinkage of the composite material can also be expressed using the degree of cure and the total relative volumetric shrinkage at the completion of curing, *V*_sh_:

(10) $$\Delta v_{r}=V_{\mathrm{sh}}\Delta \alpha$$

where *V*_sh_ is the total volumetric cure-shrinkage ratio at complete curing, and Δα is the change in the degree of cure relative to the selected reference state. Both quantities are dimensionless. Thus, the chemical shrinkage strain of the composite material during the curing process is

(11) $$\varepsilon_{0}^{r}=1-\sqrt[{3}]{1-V_{\mathrm{sh}}\Delta \alpha }$$

This homogenized treatment does not capture local variations in filler content and may therefore miss spatial differences in shrinkage and residual stress caused by alumina sedimentation.

The strain evolution can be divided into three stages. In Stage I, the composite material remains in a viscous flow state. Before gelation, thermal deformation and chemical shrinkage are largely accommodated by viscous flow, and little mechanically retained stress is generated. In Stage II, after the material reaches the gel point, both the thermal strain caused by thermal expansion and the chemical shrinkage strain caused by the curing reaction emerge simultaneously. Meanwhile, the thermal strain increases slightly after reaching the gel point and gradually stabilizes as the temperature remains largely unchanged. Stage III is the cooling stage. The curing reaction has ended, and the curing shrinkage caused by chemical shrinkage is complete [39]. This simplified stage division assumes that effective stress accumulation begins near gelation, although gelation is gradual and depends on the criterion used to define the gel point. It also does not explicitly account for vitrification, which progressively restricts molecular mobility and stress relaxation and may therefore shift the predicted onset of stress locking.

Based on the study of the overall first principal stress at various stages during the curing process of the tri-post insulator, it can be concluded that at 90 min, the material has reached gelation but still has a relatively low degree of cure and modulus, resulting in low overall stress concentrated mainly near the insulator–sleeve bonding edge. At the end of curing (880 min), cure shrinkage and cooling increase the maximum first principal stress to 58.9 MPa, with stress concentrations in the central body and near the sleeve interface. Although the stress magnitude evolves, the main concentration regions remain largely unchanged after gelation. Because the tensile strength of epoxy/Al_2_O_3_ composites is substantially lower than their compressive strength, the first principal stress is used to assess failure according to the maximum tensile stress criterion [24].

Recent studies have further combined curing simulation with data-driven process optimization. Wang et al. used a radial basis function neural network and a genetic algorithm to optimize the curing process of GIS basin-type insulators, reducing the residual strain and the non-uniformity of the degree of cure [40].

The autocatalytic reaction model can effectively describe the cure kinetics of epoxy composites used for insulators, while the coupled simulations can identify the main locations and evolution of strain concentration during curing. However, the predicted magnitudes remain sensitive to constitutive assumptions, material parameters, and boundary conditions. Embedded strain-gauge and thermocouple measurements during casting are therefore required for model calibration and validation. Comparing the measured and simulated temperature and strain histories can help refine the boundary conditions and improve the reliability of residual stress prediction.

## 3. Detection Technology

Accurately detecting the magnitude of residual stress effectively verifies theoretical models and supports structural design and process optimization. However, residual stress concentration is not a physical defect like pores or cracks; it does not react obviously to electrical, optical, or thermal fields under normal conditions, making direct evaluation using conventional defect-inspection methods difficult. For residual stress measurement, in-factory evaluation methods such as thermal-cold cycles, hydrostatic type tests, and dye penetrant inspections only indirectly reflect the influence of residual stress. They cannot accurately measure the magnitude and distribution of residual stress, nor can they achieve mass product quality inspections. Commonly used quantitative detection methods include embedded sensors, direct mechanical measurement, and indirect non-destructive testing (NDT) methods [41,42].

### 3.1. Embedded Sensors

The factory hydrostatic test only focuses on overall mechanical strength, making it difficult to achieve a numerical evaluation of the residual stress in epoxy resin insulating components. Embedded temperature sensors, strain gauges, and fiber Bragg gratings (FBGs) are often used to measure the internal temperature field of the mold cavity and the curing deformation process of the insulator. Temperature sensors provide reliable local thermal histories during curing, but they do not measure residual stress directly. The stress must be derived using a thermo-mechanical model, which introduces additional uncertainty. Chen et al. arranged 12 temperature sensors from top to bottom inside the insulator mold and found that spatial temperature field differences easily occur during the curing process. The different reaction rates of the resin system form large residual stresses locally [43]. Kyriazis et al. [44] embedded K-type thermocouples into epoxy resin to monitor the exothermic temperature field and placed resistance strain gauges at the bottom of the mold cavity to record the deformation caused by resin shrinkage. The results showed that thermocouples can accurately predict material curing, and internal curing shrinkage deformation begins after the resin reaches its gel point [44]. Dong et al. [45] embedded resistance strain gauges into low-temperature grade epoxy resin to monitor the strain response of the material in real-time during different curing processes. The results showed that different curing temperature profiles generate different curing residual strains, and samples with smaller curing residual strains exhibit higher impact strength in both room temperature and low-temperature environments [45]. Resistance strain gauges provide direct local strain measurements. However, temperature effects and local embedding conditions may bias the measured response, while discrete gauges cannot resolve the overall stress distribution.

**Figure 2:**
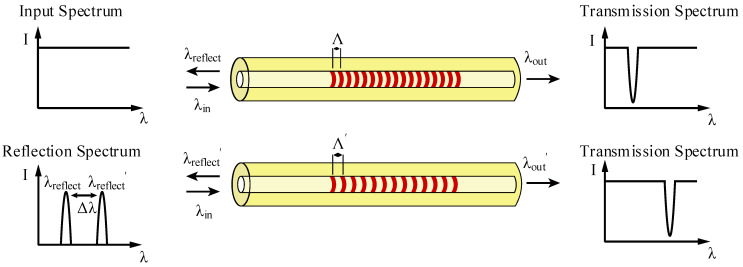
Principle of residual stress measurement using fiber Bragg grating.

As shown in Figure 2, compared with strain gauges, FBGs have the advantages of flexible arrangement, high sensitivity, and orientation [46]. FBGs offer high sensitivity and flexible placement. Nevertheless, their simultaneous response to strain and temperature requires compensation, and the measurements remain limited to predefined locations or sensing paths. During the curing shrinkage test of the insulator, placing the FBG at the central axis of the sample can reduce the measurement error caused by the radial shrinkage of the sample. Considering the effects of temperature T and strain ε on the central wavelength of the FBG, Wu et al. and Wang et al. calibrated the wavelength correction coefficient for the curing strain of the epoxy composite to determine the deformation amount of the insulator during curing [47,48]. More recently, Zhang et al. [49] developed a thermocouple-compensated FBG method for the high-temperature curing of GIS epoxy/Al_2_O_3_ composites. The method enables the in situ identification of gelation, vitrification, degree of cure, and residual strain [49].

Embedded temperature sensors, strain gauges, or FBGs can detect the temperature and deformation of epoxy-based insulators during the molding process, thereby facilitating further calculation of the residual stress magnitude. This provides good support for structural optimization, formulation selection, and process improvement. However, embedded sensors act as foreign objects in the curing system and may alter the local material response. They are therefore more suitable for studying stress evolution than for reconstructing the full residual stress field.

### 3.2. Direct Mechanical Measurement

The hole-drilling method is a high-precision, low-damage residual stress testing method, which is highly applicable for measuring near-surface residual stress in isotropic linear elastic materials [50,51]. A blind hole with radius r and depth h is drilled at any point on the insulator. The original stress equilibrium state on the surface is disrupted, generating released strain, which can be calculated using the released strain measured by a three-way resistance strain gauge. Tests on the 550 kV GIL tri-post insulator showed that tensile stress mainly manifests near the central cylinder and inserts, reaching 13.2 MPa, while compressive stress is predominant in the leg abdomen. The distribution trend is consistent with calculations, but the magnitude differs somewhat from the simulation results. This discrepancy occurs because the hole-drilling method cannot locate the interface region, and the measurement of released strain is affected by factors such as the internal molded structure and sensor precision, leading to potential errors. Nonetheless, reducing the residual stress at the central cylinder and the resin end face is necessary to improve the structural strength of the epoxy-based insulator.

### 3.3. Indirect Non-Destructive Testing Method

NDT methods mostly utilize external stimuli such as light or sound acting on epoxy resin insulators or insulating materials. The internal residual stress distribution affects their transmission processes, and the magnitude and distribution information of residual stress are obtained through inversion. NDT methods preserve the integrity of the insulator, but their quantitative accuracy depends on material-specific calibration and stable signal acquisition.

**Figure 3:**
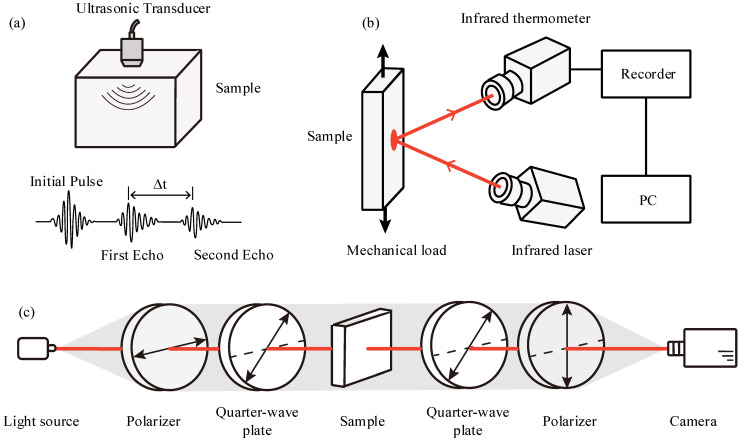
Schematic diagrams of three typical NDT principles: (**a**) ultrasonic testing, (**b**) infrared thermography, and (**c**) photoelasticity.

As shown in Figure 3, three typical NDT configurations—ultrasonic testing, infrared thermal imaging, and photoelasticity—are presented to demonstrate their respective sensing principles. The photoelasticity method is based on the anisotropic optical polarization of polymer chains, determining the magnitude of residual stress through birefringence measurement. As an NDT method, it features convenient operation and simple equipment requirements [52]. Photoelasticity provides intuitive full-field stress maps for transparent specimens. However, high alumina loading greatly reduces optical transmission. For curved insulators, refraction and optical-path variations distort the fringe pattern and make quantitative stress inversion difficult. Aung et al. [53] used the photoelasticity method to test the effect of thermal stress on the dielectric breakdown of epoxy resin, characterizing that the thermal stress near the needle electrode could reach 10 MPa. These results provide a reference for detecting residual stress in transparent dielectrics [53]. Liang et al. [54] utilized the thermoelastic method to measure the laser-induced temperature rise in epoxy/alumina composites under different mechanical stresses and reconstructed the residual stress distribution of a full-scale insulator based on the stress-temperature relationship [54]. The thermoelastic method can evaluate residual stress from the stress-dependent thermal response, but the temperature change is small and requires careful calibration [55].

The acoustoelastic method measures residual stress utilizing the linear relationship between ultrasonic wave velocity and internal stress. Compared with the hole-drilling method, strain gauge method, and photoelasticity method, it has significant advantages in non-destructive, non-invasive, and penetration capabilities [56]. When elastic acoustic waves propagate in a solid with residual stress, their velocity depends on the second-order elastic constants and density of the material, and correlates with higher-order elastic constants and stress. This relationship between stress and acoustic wave velocity is called the acoustoelastic effect. For full-scale GIS/GIL insulators, geometric complexity is a major practical limitation of ultrasonic stress measurement. Variations in the local surface normal and coupling conditions may alter the propagation path and reduce measurement repeatability [57]. Material heterogeneity caused by the non-uniform distribution of alumina may also affect the local acoustic velocity and attenuation.

For the acoustoelastic detection of residual stress in GIL/GIS epoxy resin insulating components, researchers from the South China University of Technology built a subsurface residual stress measurement system for GIS basin-type insulators based on the ultrasonic critically refracted longitudinal (LCR) wave acoustoelastic effect. Test results on a 252 kV GIS basin-type insulator showed that the compressive residual stress at a checkpoint near the center conductor was 0.38 MPa, and at a checkpoint far from the center conductor, it was 0.12 MPa. Test results on a 550 kV GIS basin-type insulator showed that the radial residual stresses were all tensile stresses and uniformly distributed, with the maximum residual stress reaching 5.8 MPa near the flange side [58,59]. Tianjin University and State Grid Tianjin Electric Power Research Institute collaboratively conducted residual stress distribution detection and imaging research based on the transmitted longitudinal wave acoustoelastic effect. Based on the spatial geometry of the basin-type insulator and mechanical arm fixed-point and delayed command controls, the cooperative debugging of the mechanical arm and the ultrasonic probe was achieved, completing the automatic detection of ultrasonic propagation velocity and residual stress at 72 points on the basin-type insulator. The residual stress distribution cloud map of the basin-type insulator indicates that the outer edge of the basin-type insulator mainly exhibits tensile residual stress, while the area around the conductor exhibits compressive residual stress. This is closely related to the pouring direction, primarily due to alumina sedimentation, curing, and cooling shrinkage of the epoxy resin mixture after pouring, with the maximum tensile stress reaching 18.10 MPa [60].

In summary, different detection methods have their respective applicable scenarios and limitations. Embedded sensors, such as strain gauges and FBGs, can be used to monitor temperature and deformation during the curing process. They are suitable for studying stress evolution mechanisms but affect local curing behavior and struggle to reflect the overall stress distribution. The hole-drilling method is suitable for quantitatively testing near-surface local residual stress, but it is slightly destructive and difficult to cover complex structures. NDT methods offer important advantages for stress-field evaluation, but each method is subject to specific constraints. Photoelasticity is limited by optical accessibility, thermoelastic measurements rely on weak thermal responses and accurate calibration, and ultrasonic methods are sensitive to material heterogeneity and propagation-path variations. For large GIS/GIL insulators with complex geometry, these factors make full-field quantitative measurement particularly challenging.

For UHV epoxy-based insulators, current methods such as the hole-drilling method and LCR wave method are suitable for near-surface detection. Transmitted longitudinal waves can be used for two-dimensional distribution measurements in regular areas. Future work should focus on overcoming the matching issue between probes and curved structures, improving ultrasonic penetration capabilities, and simultaneously promoting the development of automated detection devices to enhance factory inspection efficiency and accuracy.

Table 2 summarizes the reported measurement performance, destructiveness, and applicability of the main residual stress measurement methods to curved and opaque epoxy-based insulators. The listed accuracy or error values were obtained under specific experimental conditions. In practice, measurement accuracy is also affected by material composition and heterogeneity, specimen geometry, calibration procedures, and other experimental factors. Therefore, for highly filled and geometrically complex GIS/GIL insulators, the practical applicability of each method should be evaluated together with its nominal accuracy.

## 4. Molding Reliability

### 4.1. Filler Sedimentation

**Figure 4:**
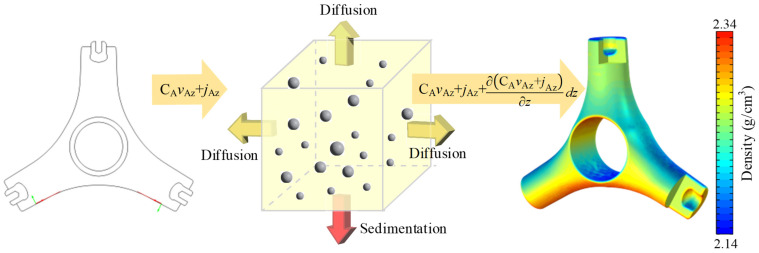
Schematic diagram of the alumina sedimentation model.

The epoxy-based insulator is cast from a mixture of epoxy resin, anhydride curing agent, and α-micro alumina [61]. During the actual manufacturing process, the preheated epoxy resin and curing agent have a low viscosity. Furthermore, their cross-linking reaction takes time, reaching the gel point in about 1 h at 140 °C. This causes the alumina particles to continuously settle toward the bottom of the mold under gravity during the initial casting stage, as shown in Figure 4. Consequently, the density of the finished epoxy-based insulator varies due to different alumina contents at different locations [62]. Therefore, alumina sedimentation should not be regarded only as a density non-uniformity problem. It also introduces a spatial gradient in filler content, which may further change the local resin fraction, curing shrinkage, thermal conductivity, elastic modulus, and thermal expansion behavior of the epoxy/Al_2_O_3_ composite.

Zheng et al. used ultrasonic non-destructive testing and found that gravity sedimentation of alumina fillers causes significant density differences inside a 126 kV tri-post insulator, with the maximum density difference between the top and bottom reaching 0.21 g/cm^3^ [63]. During solid particle sedimentation, the movement speed of each particle is the key factor affecting the final sedimentation degree of the insulator, which is related to particle diameter, density, and liquid viscosity [64]. Kim et al. proposed a photoacoustic method to evaluate alumina volume fraction in epoxy/alumina composites and achieved a measurement error below 5% for filler volume fractions up to 50% [65]. Researchers from Tianjin University established a three-dimensional physical model of alumina sedimentation in tri-post insulators based on Stokes’ law and verified the simulation results by slicing density measurements. Their results showed that the density difference between the upper and lower sides of the conducting rod in a 550 kV GIL tri-post insulator reached 0.13 g/cm^3^, while the maximum overall density deviation during forward casting reached 0.202 g/cm^3^ [66]. Fei et al. further found that the mass fraction difference in the alumina filler around the inserts could reach 11.07%, and increasing the initial casting viscosity can effectively suppress filler sedimentation [67].

Since sedimentation directly changes the local Al_2_O_3_ content, studies on epoxy systems with different filler contents are useful for understanding residual stress formation. Hao et al. investigated the curing shrinkage and residual strain of epoxy resins with different Al_2_O_3_ contents using a thermocouple-compensated fiber Bragg grating method and DSC tests [68]. They found that Al_2_O_3_ filler can prolong the gel time, reduce the curing exothermic peak temperature, and change the residual strain and glass transition temperature of the epoxy system. This indicates that the local filler content can affect both the curing reaction and the post-gel shrinkage behavior. Zhang et al. further confirmed that residual strain, degree of cure, gelation, and vitrification are key parameters for evaluating the curing characteristics and formulation optimization of GIS epoxy/Al_2_O_3_ composite insulation materials [49].

The alumina–epoxy interface also affects stress transfer and fracture behavior. Because rigid alumina particles and the epoxy matrix have different elastic moduli and thermal expansion behaviors, local deformation incompatibility can occur around the particle–matrix interface during curing, cooling, and mechanical loading. Tang et al. [69] investigated the three-dimensional fracture behavior of epoxy–alumina composites using micro-CT, digital volume correlation, and linear elastic fracture mechanics. Their results showed that cracks often initiate from the matrix–particle interface near the notch tip, which was attributed to particle-induced stress concentration [69]. Zhang et al. reported that weak interfacial bonding between inorganic Al_2_O_3_ particles and the epoxy matrix can lead to poor stress transfer, whereas silane modification improves interfacial compatibility and mechanical properties [70].

Overall, alumina sedimentation affects epoxy-based insulators through both macroscopic filler-content gradients and microscopic interfacial effects. At the macroscopic scale, non-uniform alumina distribution changes local shrinkage, thermal conduction, modulus, and thermal expansion, causing residual stress redistribution under the constraints of the conductor, insert, sleeve, and mold. At the microscopic scale, the alumina–epoxy interface transfers stress between rigid particles and the polymer matrix, but it may also become a preferential crack-initiation region under local tensile stress. Therefore, filler sedimentation can simultaneously influence local material properties, residual stress distribution, stress concentration, and crack initiation, and it also increases the difficulty of residual stress measurement because the acoustic, optical, and mechanical responses become spatially non-uniform.

### 4.2. Mechanical Reliability

Residual stress affects the mechanical strength and fatigue performance of epoxy resin composites, increasing the probability of warpage and in-service faults. Such faults may include transverse cracking, brittle fracture, and reduced fatigue life, which are considered crucial causes of insulation failures under operating conditions [71].

For insulators with good sealing, such as basin-type insulators and disc-type insulators, the hydrostatic burst test is a verification method to evaluate the influence of residual stress on insulator reliability [72]. Gao et al. [73] conducted a simulation study on the stress distribution of a single-phase basin-type insulator under hydrostatic test conditions, locating the stress concentration areas in high-curvature regions. Based on this, they proposed design suggestions to optimize the insulator structure by reducing the surface curvature [73]. Guo et al. [74] analyzed the strain changes in basin-type insulators during hydrostatic tests. The results demonstrated that the simulation results agreed well with the strain measurement results based on fiber Bragg gratings, and the location of the first principal stress concentration was the initiation point of the insulator fracture [74].

Multi-physics simulation is also an effective method for studying the stress distribution and mechanical reliability of insulators. Kang et al. [75] conducted a multi-field coupled simulation study of electromagnetics, temperature, fluid, and stress on a 550 kV GIL basin-type insulator under operating conditions. They analyzed the effect of the temperature dependence of key physical and chemical parameters of the epoxy composite on the stress evolution of the insulator, locating the thermal stress concentration points at the triple junctions and concave areas. Furthermore, using the penalty function method and the Mooney-Rivlin model, they demonstrated the effectiveness of a semi-conductive rubber buffer layer in improving the interfacial stress distribution [75]. Dong et al. [76] built a multi-physics simulation model of a GIL tri-post insulator under comprehensive electromechanical stress based on energy theory. The results showed that asymmetric mechanical loads cause an extremely uneven axial stress distribution in the insulator, leading to an increased failure probability. Adopting a horizontal laying method and specific post-installation angles can effectively suppress stress concentration and enhance mechanical reliability [76]. The literature [77] parameterized the typical positions of the insulator and improved the insulator structure by combining decision variables and a genetic algorithm. They further simulated and analyzed the electric field distribution and the stress distribution of the insulator under transport and vertical installation states before and after optimization. The proposed optimization strategy increased the insulation margin by 6.3% without compromising the mechanical reliability of the insulator.

Currently, there is little research evaluating the mechanical reliability of epoxy-based insulators considering the thermodynamic property parameters and the spatial distribution of residual stress caused by filler sedimentation. Therefore, multi-physics studies of insulators coupled with residual stress distribution are of great significance for analyzing the mechanical reliability margin of insulators. The research team at Tianjin University investigated the distribution rules of tensile and compressive stresses in a 550 kV GIL tri-post insulator at different life cycle stages. They found that the coupled tensile stress from wedge ring assembly and residual stress reached 61.2 MPa during the factory stage, explaining the frequent interfacial cracking between the sleeve and resin in the factory. During operation, the tensile stress concentration becomes more severe under the influence of temperature gradients and conducting rod gravity, reaching a maximum of 66.36 MPa. Damage fracture simulations and model tests under radial loads have proven the applicability of conducting insulator reliability analysis based on tensile strength distribution. Based on the Rankine criterion, its mechanical reliability was evaluated. Under the influence of residual stress, the maximum failure risk coefficient during steady-state operation of the insulator reached 0.82. Therefore, special attention must be paid to filler sedimentation and residual stress issues during the molding process [39].

Existing models have simulated and analyzed the principal stress and mechanical reliability during the factory and installation processes. For the actual operating conditions of epoxy-based insulators, the influence of conditions such as inclined laying, vertical laying, and assembly misalignment should also be considered to analyze and calculate whether the mechanical reliability of the insulator meets the requirements under special circumstances. Combined with suppression tests on insulator filler sedimentation, this will further improve the safety margin of subsequently commissioned insulators. For industrial implementation, residual-stress control should be integrated into manufacturing and factory quality-control procedures. Formulation-specific material parameters and temperature or strain measurements during curing can be used to calibrate numerical models and identify abnormal process conditions. For factory inspection, rapid non-destructive screening may be combined with local quantitative measurements at critical regions. Before routine application, the method should be evaluated in pilot production and on full-size insulators. These tests should confirm that the results are repeatable, the inspection time and cost are acceptable, and the method can be integrated into the existing production process. Clear calibration procedures, reporting rules, and acceptance limits are also required.

## 5. Conclusions

(1) The exothermic cross-linking reaction between the epoxy resin and anhydride causes the internal temperature of the epoxy-based insulator to exceed the process temperature. During the high-temperature curing stage, the strain is mainly dominated by chemical shrinkage, and the material modulus increases as the degree of cure increases. During the cooling stage, thermal shrinkage causes the epoxy-based insulator to further displace inward. This exacerbates the stress concentration at the boundary between the sleeve and the insulator.
(2) Current research and application status indicate that existing detection methods are more suitable for comparing the suppression effects of process improvements and structural optimizations on residual stress. Detecting the absolute value of residual stress alone is extremely difficult and requires precise calibration, parameter detection, and calculation. The residual stress distribution of epoxy-based insulators possesses spatial attributes, including both amplitude and location information. Future work should focus on the research and development of automated detection devices and spatial inversion technologies.
(3) The impact of residual stress on mechanical reliability can be indirectly evaluated through methods such as model coupling, mechanical loading, and thermal-cold cycles. However, with a deeper understanding of burst and disintegration failures under actual operating conditions, the influence of residual stress on insulation performance deserves further in-depth study.

## Figures and Tables

**Table 1:** Fault Types of Epoxy-based Insulators.

No.	Fault Type
1	Burst failure—Single leg fracture
2	Burst failure—Fracture from the epoxy/insert interface of the lower-left leg to the epoxy/conductor interface
3	Burst failure—Partial fracture at the epoxy/insert interface
4	Burst failure—Single leg fracture
5	Burst failure—Epoxy resin detachment at the top of the leg
6	Burst failure—Single post burst of the sliding tri-post insulator

**Table 2:** Comparison of Residual Stress Measurement Methods.

Measurement Method	Reported Measurement Performance Under Specific Test Conditions	Destructiveness	Suitability for Curved Surfaces	Suitability for Opaque Materials
Embedded temperature sensor	Class A tolerance: ±(0.15 + 0.002|t|) °C	Intrusive	Good for predefined points	Good
Resistance strain gauge	±1 to 5 με in normal conditions, up to ±5 to 10 με in complex field conditions	Intrusive	Limited	Good
FBG sensor	Typical strain sensitivity: approximately 1.2 pm/με; hysteresis ≤ 0.058%; repeatability ≤ 0.045%	Minimally intrusive	Good	Good
Hole-drilling method	For a specific Ti6Al4V specimen: 14–62 MPa uncertainty; 6–22% relative uncertainty	Semi-destructive	Limited	Good
Photoelastic method	Generally within 5% for transparent planar specimens; deviations at some points exceeded 5% when the fringe order *N* ≤ 2, whereas they were generally below 5% when *N* ≥ 2.5	Non-destructive	Limited	Poor
Thermoelastic method	For epoxy/Al_2_O_3_ insulators: Approximately 4 MPa; relative error < 10%	Non-destructive	Moderate	Good
Acoustoelastic method	When using critically refracted longitudinal wave method: 0.69 MPa; principal-stress direction error 2.97°	Non-destructive	Moderate	Good

## Data Availability

No new data were created or analyzed in this study. Data sharing is not applicable.
